# Current Status of HIV/AIDS in South Asia

**DOI:** 10.4103/0974-777X.56249

**Published:** 2009

**Authors:** Chaturaka Rodrigo, Senaka Rajapakse

**Affiliations:** *University Medical Unit, National Hospital of Sri Lanka*; 1 *Department of Clinical Medicine, Faculty of Medicine, University of Colombo, Sri Lanka*

**Keywords:** HIV/AIDS, South Asia, HAART

## Abstract

**Background:**

According to the United Nations Joint Program on HIV/AIDS, 33.2 million adults and children are living with the infection worldwide. Of these, two to three million are estimated to be in South Asia. All countries of the region have a low prevalence of human immunodeficiency virus (HIV). However, it is important to review the current epidemiological data to identify the trends of infection as it would have implications on prevention.

**Materials and Methods:**

We performed a MEDLINE search using phrases ‘South Asia’ plus ‘HIV’, ‘AIDS’, and names of individual countries in South Asia (limits: articles published in last 10 years, in English language). Clinical trials, reviews, meta-analyses, letters, editorials, and practice guidelines were all considered. The following countries were included as belonging to South Asia; Afghanistan, Bangladesh, Bhutan, India, Maldives, Nepal, Pakistan and Sri Lanka. Recent estimates and data on country status, and details of national control programs were obtained from websites of international agencies such as the World Bank and United Nations Joint Program on HIV/AIDS (UNAIDS).

**Results and Discussion:**

This review looks into many aspects of HIV infection in South Asia including country profiles with regard to infection, economic and psychological burden of illness and treatment issues in the South Asian context.

## INTRODUCTION

Infection with human immunodeficiency virus (HIV) and subsequent development of acquired immunodeficiency syndrome (AIDS) poses a significant challenge to modern medicine and humanity. According to the United Nations joint program on HIV/AIDS (UNAIDS), currently there are 33.2 million adults and children living with HIV/AIDS. The highest number of patients is reported from sub-Saharan Africa.[1]

Outside Africa, Asia remains a potential breeding ground for an epidemic. Given the massive population density, an epidemic in India and China will have a huge impact on the global economy and human survival similar to that of sub-Saharan Africa.[2] It is estimated that in 2007 there were 4.9 million people with HIV/AIDS in Asia, with 440,000 new infections.[2] Although heterosexual intercourse is considered the main risk behavior for spread of HIV in Africa, in Asia it is intravenous drug use (IVDU).[3] However, in South Asia, transmission via sexual contact is predominant.[4] All countries in the South Asian region are still considered to have a low prevalence of HIV, though numbers are increasing in Pakistan and Nepal.[5] There are many risk factors in the region favoring an epidemic of HIV, such as illiteracy, poor economic status, poor sanitary and health facilities, social taboos on discussion of sex and malnutrition. The high prevalence of tuberculosis in the region will play a significant role in reducing life expectancy should HIV/AIDS rates rise.[6]

## MATERIALS AND METHODS

We performed a PUBMED search using phrases ‘South Asia’ plus ‘HIV,’ ‘AIDS,’ and names of individual countries in South Asia (limits: articles published in last 10 years, in English language). Clinical trials, reviews, meta-analyses, letters, editorials, and practice guidelines were all considered. The following countries were included as belonging to South Asia; Afghanistan, Bangladesh, Bhutan, India, Maldives, Nepal, Pakistan and Sri Lanka. The software endnote X1.01 was used to filter articles.

We read through the abstracts and selected relevant articles from 1346 search results. Coding was done by two reviewers independently blinded to each other. Sources were screened for a well described methodology, rigor of statistical analysis and an adequate sample size where relevant. Recent estimates and data on country status and details of national control programs were obtained from websites of international agencies such as the World Bank and the UNAIDS. The inter-reviewer agreement for data included in the final synthesis was 100%.

This paper looks into many aspects of HIV infection in South Asia, including viral epidemiology, country profiles with regard to infection, economic and psychological burden of illness, and treatment issues.

## CURRENT STATUS OF HIV/AIDS IN SOUTH ASIA

### Viral epidemiology

There are two major types of HIV causing infections in humans, HIV-1 and HIV-2. The majority of infections in South Asia are by HIV-1. However there are confirmed cases of HIV-2 infection in India and Nepal though numbers are significantly less than HIV-1.[7]

Depending on genetic variation, HIV −1 viruses are divided in to 3 groups (M= major, O= outlier, N= non-M and non-O).[7] Human infections are mainly by M group viruses which are further subdivided in to nine subtypes or ‘clades’ (A-D, F-H, J and K). One or more subtypes may predominate in a given geographic area. For example, the majority of HIV-1 infections in India are by subtype C. Subtypes are not classified in groups N and O.[7]

If there is more than one circulating subtype in an area, new recombinant forms can arise from co-infection or super-infection. If such a recombinant form is identified from three epidemiologically unlinked people, it is designated as a circulating recombinant form (CRF).[7] Isolation of such a form in epidemiologically linked people makes it a unique recombinant form (URF).[7] URFs as opposed to CRFs have not shown evidence of epidemic spread.Table 1 shows isolated subtypes and CRFs from infected individuals in some South Asian countries.[8]

**Table 1:** Subtypes and circulating recombinant form reported in India, Bangladesh and Nepal[8]

Country	Subtype	Subtype distribution (%)
India	C	92.5
	B	3.7
	BC	1.9
	A	1.3
	Other	0.7
Bangladesh	C	86.5
	A	3.8
	01_AE	2.9
	G	1.9
	B	1.9
	U	1.9
	07_BC	1.0
Nepal	C	100

### Country profiles

#### India

Outside Africa, India has the highest number of people living with HIV and AIDS.[1] According to UNAIDS country fact sheet 2008 update, the total number of adults and children estimated to be living with HIV and AIDS in India was 1.8 – 3.2 million with prevalence rates for adults estimated to be within 0.2 - 0.5%.[9] It is also estimated that currently 0.67 to 1.2 million adult women are living with the disease.[9] These figures have declined when compared with data of 2003 and 2005, indicating a positive impact of prevention and control programs.

HIV surveillance was established in 1985-86 in India. Evidence of HIV infection in female commercial sex workers (CSWs) in southern Indian states surfaced at that time. In 1989, HIV was also reported from IV drug users in Manipur state.[10] India's risk of HIV transmission is not uniform throughout the country or its population. There are six high prevalence states, namely; Tamil Nadu, Karnataka, Maharashtra, Andhra Pradesh, Manipur and Nagaland.[11] Even within the states there are high prevalence pockets. The high risk groups include female commercial sex workers (CSW), IV drug users and men having sex with men (MSM).[11]

The National AIDS Control Organization (NACO) was set up in 1992 to carry out HIV/AIDS prevention, control and surveillance programs more effectively. The agenda of NACO was set in three phases.[11] The first phase (1992–1999) concentrated on blood safety, high risk groups, increasing awareness and improving surveillance. The second phase which ended in 2006 concentrated on giving more responsibility to states with involvement of state departments and non governmental organizations in prevention, targeted interventions for high risk groups and general interventions for public.[11] Phase 3 of the program involves building on the foundations of phases 1 and 2 towards more effective measures of prevention and control.[11]

The traditional and official view of India is that the sex industry and especially the CSWs are responsible for the majority of HIV transmissions. NACO estimates that 86% of transmissions are due to sexual risks, 2.4% due to IVDU, 2.0% due to receipt of blood/blood products and 3.6% due to perinatal transmission.[12] Currently there are an estimated 0.83 – 1.2 million female CSWs in the country.[13] India is unique in the sense that MSM is a common occurrence compared to other countries in the region though the numbers of male CSWs are obscure. In a prospective study conducted in Pune, 6.6% out of 10,800 men attending three STD clinics admitted to having sex with other men.[10] In two high HIV prevalence areas of India, Mumbai and Chennai, prevalence rates of 9.6% and 6.8% for HIV were reported in MSM respectively.[10]

#### Pakistan

Pakistan reported its first few cases of HIV at the same time as India. By 2001 HIV was reported in many parts of the country.[14] Pakistan is still considered to be a low prevalence country for HIV. Approximately 96,000 people are currently living with the infection with a prevalence rate around 0.1%. The identified high risk groups include; female CSWs and their clients, long distance truck drivers, MSM, recipients of paid blood donors and IVDUs.[14] The number of adult women living with infection is estimated to be between 19,000 – 42,000. Estimated number of deaths due to HIV was between 3500 – 8200 in 2007.[15] The official view of Pakistan is that sexual risks due to prostitution are the predominant mode of transmission with MSM also playing a significant role. Illiteracy and lack of awareness of safe sex practices encourage transmission of HIV and other sexually transmitted infections.[16] Overall contraceptive prevalence by 2007 was 27.6% with only 5.5% using condoms.[15]

The government initiated the National AIDS Program (NAP) in 1987 to coordinate the efforts of tackling the threat of HIV. In 2001, a national HIV/AIDS strategic framework was developed to address the issues of prevention and control in Pakistan.[16]

IVDU is a significant problem in Pakistan with an estimated 100,000 IVDUs having an HIV prevalence of 10-50% within them (data from four urban areas).[16] Unsafe blood transfusion is another important problem; in some estimates approximately 40% of annual blood transfusions are not screened for HIV (although UNAIDS maintains that 87% of donated blood units are screened for HIV in a quality assured manner).[15]

A multitude of socio-cultural factors including political tension, religious restrictions on discussion of sex, social stigma and extremism limit the accessibility of prevention and control programs to a sizable population.[16] Influx of refugees and migrants into these communities increases the risk of exposure to HIV, and surveillance data for the country may underestimate the true prevalence of disease due to non-availability of data.

#### Sri Lanka

Sri Lanka is considered to have a very low prevalence of HIV. According to UNAIDS estimates, 3800 Sri Lankans were living with HIV by end of 2007. Prevalence is estimated to be less than 0.1%.[17] Altogether there have been 957 reported cases of HIV so far with 177 confirmed dead.[18] The ratio of HIV positive men to women is 1.4:1. Majority of patients are reported from the capital Colombo and its suburbs.[18] Interestingly, the proportion of women with HIV has risen from 21% to 42% between 1991 and 2007.[18] Approximately 1400 women are currently living with HIV in the country.[17]

The predominant mode of transmission in Sri Lanka is heterosexual intercourse with female CSWs playing a major role.[18] Estimates of numbers of female CSWs in the country vary from 5000 – 50,000 in different studies.[18] Still, the prevalence of HIV among female CSWs is considered to be very low. Displacement of communities due to internal military conflict in the North and East of the country, and migration of women for work are potential risk factors for sexual abuse and HIV transmission. IVDU is much less common in Sri Lanka with only an estimated two per cent of opioid drug users injecting drugs.[18] Only one IVDU has tested positive for HIV so far.[18]

Cultural factors limiting open discussion of sex, lack of awareness of safe sex in rural areas and high risk groups pose a problem for preventive programs. Though overall contraceptive prevalence stands at an impressive 70%, the prevalence of condom use is 3.7%.[17]

The government of Sri Lanka took the initiative in controlling HIV and other sexually transmitted diseases by establishing the National Sexually Transmitted Diseases and AIDS control program (NSACP) in 1992. The NSACP coordinates the HIV/AIDS prevention and control programs in liaison with other government ministries, departments and non-governmental organizations. In addition to the existing sero surveillance system, a second generation behavioral surveillance was started in 2006 amongst high risk groups.[18]

#### Bangladesh

Bangladesh is also considered a low prevalence country for HIV. Its first case of HIV was reported in 1989.[19] Currently there are 1207 HIV infections reported with an overall prevalence of 0.1%.[20] The total number of people with AIDS is 365 of which 123 had died.[20] It is estimated that around 12,000 people are living with HIV in Bangladesh.[21]

The government of Bangladesh was actively involved in tackling the problem as early as in 1985 by setting up the National AIDS Committee (NAC). The National STD/AIDS Program (NSAP) is the main body responsible for policy formation, information provision, regulation and coordination of HIV/AIDS related activities.[22] A national strategic plan was initiated by the government of Bangladesh in two phases. The first phase was from 1997 – 2004 and the second phase started in 2004 and is expected to run until 2010.[22]

Prevalence of HIV is monitored by the national HIV surveillance system annually. Since it is a low prevalence country, surveillance is more focused on most at risk persons (MARPs).

While the overall prevalence for HIV is low in general population, it is high amongst IVDUs and currently stands at 7% (in Dhaka).[21] Models based on current data and risk behaviors predict that, if uncontrolled, an HIV epidemic can arise amongst IVDUs that will later ‘spill over’ to sex workers.[22] Behavioral surveillance surveys and a cohort study involving both HIV positive and negative male IVDUs have shown that needle sharing is still a problem despite having a needle exchange program in place. Having access to a sterile needle at time of injection was the main factor determining sharing.[23] In another cohort involving 135 female IVDUs, needle sharing rate was higher and 63.1% also sold sex, thus increasing risk for transmission.[24]

HIV prevalence amongst male and female sex workers had remained low according to national surveillance system and currently stands at 1%.[21] However, behavioral surveillance revealed that high risk behavior such as group sex and anal sex are common in this group.[22] The overall condom use is not satisfactory (less than 15%) and even if a condom is used, many have not used it correctly.[22]

Returnee migrant workers and their spouses are another high risk group. Of 371 HIV infected patients attending a special clinic, 54.2% were returnee migrants.[22] A separate study carried out on traveling workers has revealed that a majority had sex with a commercial sex worker, and the rate of condom use was low.[25]

#### Nepal

The scenario in Nepal is more dangerous in view of an impending epidemic in comparison to other countries. As in Bangladesh, the overall prevalence of HIV in general population is less than one per cent. However, the prevalence in high risk groups remains alarmingly high.[26] The first case of HIV was reported in Nepal in 1988. By 2008 around 1750 cases of AIDS and around 11,000 cases of HIV had been reported.[26] UNAIDS estimates that between 50,000 to 100,000 adults and children are currently living with HIV in Nepal.[27] Chander and Pahwa in 2004 reported the presence of HIV - 2 infections in Nepal.[28] High risk groups in Nepal include female CSWs, IVDUs, MSM and migrant workers.

In 2002, the country was considered to have an overall HIV prevalence of 0.3%, but the value was alarmingly high for IVDUs (40%) and female CSWs (20%) in Kathmandu.[29] However, there have been questions about the validity of serological and surveillance methods that recorded such high rates in these groups.[29] Further, these two groups account for less than one per cent of the workforce (adults between 15 -49) of Nepal and are clustered in major cities and towns.[30]

Cross border trafficking of sex workers is a major problem contributing to increasing HIV rates in both India and Nepal. The traditional view of HIV coming to Nepal from India is challenged now with more emphasis on a two way flow model.[30] It is estimated that 5000 – 7000 Nepali women are taken across the border to India annually in sex trafficking and currently there are around one to two hundred thousand Nepali women in Indian brothels.[30] In a study by Silverman *et al.* published in 2008, of 246 sex trafficked Nepali women, 74 (30.1%) tested positive for HIV while 48 (20.4%) had syphilis and hepatitis B was detected among eight of 210 (3.8%).[31] In a survey involving 580 sex workers in Eastern India, Sarkar *et al.* showed that the HIV positivity was significantly higher in Nepali women (43%) when compared to Bangladeshi (7%) or Indian sex workers (9%).[32]

HIV among MSM is estimated to be around 3.3% and rising (Data from Katmandu valley). As the total number of MSM community is estimated to be anywhere from 64,000 to 193,000, it is also a group needing specific targeted measures on prevention.[26]

Drug and sex trade are intimately linked and there is evidence for flow of infection between these high risk groups.[30] HIV infection rate amongst IVDUs is estimated to be between 22 – 35% in various studies. In Katmandu region, it is estimated to be as high as 50%. However, this remains a problem in isolated pockets such as Katmandu, Pokhra and eastern border areas.[33,34]

The first national AIDS prevention and control program was started in 1988 by government of Nepal. A National AIDS Coordinating Committee (NACC) was set up in 1992. The NACC now reports to a more recently set up unit, National AIDS Council (NAC).[26]

After hostility ended between Maoist guerillas and government forces, there is more freedom to access rural areas and townships for prevention programs. The fallout of a decade of hostilities and social disorganization in relation to spread of HIV is yet to be determined. Some authors predict a rise in HIV prevalence as more migrants and sex workers return to Nepal with political stability.[30]

#### Maldives, Bhutan and Afghanistan

Maldives is another low prevalence country. The first inland case of HIV (a foreigner) was reported in 1987, but the first Maldivian national with HIV was reported in 1991.[35] By 2003, 135 cases of HIV were reported in the islands (123 foreigners, 12 natives).[35] Risk factors for HIV in Maldives include, high mobility of population, high rates of divorce and remarriage, tourism, immigrant workers and a too dispersed population (in 200 inhabited islands) making logistical problems in having access to control programs.[35] Maldives has its own HIV control program coordinated by National AIDS Council (started in 1987). The awareness programs have been highly successful with more than 99% of households being aware of HIV/AIDS.[35]

Data on HIV infection in Bhutan is scanty. The World Bank country report on HIV/AIDS states that 144 HIV positive people have been reported since 1993. About 500 people are estimated live with HIV and the sentinel surveillance data in 2007 place the overall country prevalence well below one per cent.[36] Female CSWs, MSM and IVDUs are high risk groups. Unfortunately data on the exact numbers of these groups or their HIV prevalence is not available. Bhutan is said to have a more casual approach towards sex than other countries in the region. However, the prevalence rates for other sexually transmitted infections (STI) also remain low.[36] A National HIV/AIDS Control Program (NACP) has been functioning since 1988 under the Ministry of Health. Strong political commitment, open discussion on sex / STIs and adequate funding by donor agencies are seen as positive factors in control of HIV/AIDS in Bhutan.[36]

Data on HIV infection in Afghanistan is also scarce and may be underestimated. By 2008, 478 cases of HIV were reported and 1000-2000 people were estimated to live with HIV. Overall prevalence is estimated to be below 0.5%.[37] Risk factors for transmission of HIV in Afghanistan include; IVDUs, unsafe paid sex, large population of displaced persons and illiteracy.[37] Afghanistan is estimated to have one million drug users with 19,000 injecting drugs intravenously.[37] HIV prevalence amongst IVDUs stands at three per cent in Kabul and the common viral subtype infecting these patients is identified as CRF 35_AD.[38] HIV/ AIDS prevention efforts have started very late in Afghanistan. The Ministry of Public Health has come out with a national strategic plan to be executed over four years starting from 2006. Overall, the country has many other health issues to tackle such as high maternal mortality, high childhood mortality and deaths from curable infectious diseases. Therefore HIV/AIDS control is not a priority for the Afghan government at the moment.[37]

A summary of HIV related details for South Asian countries are given in Table 2.

**Table 2:** Virology, epidemiological patterns, risk groups and details of control programs for HIV in South Asian countries[1,9,11,15,16,17,18,21,26,27,35,36,37,43]

	India	Pakistan	Nepal	Bangladesh	Sri Lanka	Bhutan	Afghanistan	Maldives
Virology	HIV1, HIV2	HIV 1	HIV1, HIV2	HIV 1	HIV 1	HIV 1	HIV 1	HIV 1
First case reported	1985	1985-86	1988	1989	1987	1993		1987
Prevalence % (year)	0.2-0.5 (2008)	0.1 (2008)	0.4 – 0.6 (2007)	0.1 (2008)	<0.1 (2007)	Low prevalence	<0.5% (2008)	N/A
Total number living with HIV (year)	1.8-3.2 M (2008)	96,000 (2008)	50,000 – 100,000 (2008)	12,000 (2007)	3800 (2007)	500 (2007)	1000 – 2000 (2008)	Not known (135 cases reported by 2003)
Number of women with HIV (year)	0.67-1.2M (2008)	19,000 – 42,000	12,000 – 17,000 (2008)	N/A	1400	N/A	N/A	N/A
Number of deaths (year*)	NK	3500 – 8200 (2007)	5000 (2007)	N/A	N/A	N/A	N/A	N/A
Risk groups	FCSW, MSM, IVDU	FCSW, MSM, IVDU	FCSW, MSM, IVDU	FCSW, MSM, IVDU, migrant workers	FCSW and their clients, migrant workers, refugees	Armed forces, prisoners, FCSW	Refugees, IVDU, FCSW	migrant workers
National control program (year established)	National AIDS control Organisation (1992)	National AIDS Program (NAP) (1987)	National AIDS Council (NAC)	The National STD/AIDS Program (NSAP)	National Sexually Transmitted Diseases and AIDS control program (NSACP)	National HIV/AIDS Control Program (NACP)	National Strategic Plan, Ministry of Public Health (2006)	National AIDS Council (1987)
Trends in incidence	Decreasing	Increasing	Increasing / Static	Increasing	Static	Not known	Not known	Not known

* Indicates number up to the year given in brackets, M - Million, NK – Not known

## BURDEN OF HIV/AIDS

The burden of HIV can be categorized as clinical, financial, psychological and social. It can be also classified as burden to patient, family and community. Discussing the clinical burden of HIV is beyond the scope of this paper. However, we intend to discuss briefly the financial, psychological and social impact of the disease with relevance to South Asian context.

### Financial

The financial impact of the disease can be assessed at two different levels: at level of individuals / family and at national level. Of the two to three million people living with HIV/AIDS in South Asia, many are in the age group of 15-49 years. The economic loss involves the loss of production due to illness while the person is alive, loss of production due to his lost life years plus costs of treatment. A study in India in mid 90s has shown that average treatment cost of a patient with HIV is twice the per capita gross national product despite exclusion of expenses for retroviral therapy.[39] With the availability of highly active anti retroviral therapy (HAART), HIV has become a chronic illness with improved longevity. One can argue that though the cost of drugs is high the fact that a person remains productive for a longer period may partially offset the cost to community. However, it is difficult to assume that an infected individual will be as productive as a healthy person.

In a study in India, on average, 43 working days were lost per patient per six months due to illness related issues. It is interesting to note that economic loss due to premature death or loss of productivity of infected population is estimated to be 10 times more than their annual treatment costs.[39]

In a paper discussing the economic burden of HIV in India, Kumarasamy *et al.* describe the state of families with more than one member infected. As cost of treatment is an issue for an average low income family, who gets priority for treatment? It is usually the breadwinner and then the children. Women become a disadvantaged group.[40]

Poverty and HIV have a two-way connection. HIV causes poverty and poverty makes people vulnerable to HIV. Studies have shown that people with poor socioeconomic background are less likely to use condoms correctly and sex workers from the lower end of the market (streetwalkers) are less capable of insisting that their client wears a condom during intercourse.[41]

We do not see major economic consequences as those seen in high prevalence countries in sub-Saharan Africa in South Asia (distress sales of land, loss of highly skilled personnel, significant reduction in gross national productivity, loss of manpower, etc). Arndt and Lewis have shown that over the period from 2000 to 2010, the annual rate of growth of GDP in South Africa would be substantially lower in comparison to a no-AIDS scenario.[42] If the infection is not controlled, South Asia will face similar consequences in future.

### Psychological

The relationship between psychiatric conditions and HIV can be summarized as; psychological reactions to a diagnosis of HIV, disorders due to the organic pathology of HIV virus/immunodeficiency (malignancies, opportunistic infections), psychological reactions to drugs, hospitalization, treatment and psychological impact of a long term fatal illness. It is also known that psychiatric illnesses increase vulnerability to HIV.[43]

There are many acute psychological reactions associated with diagnosis of HIV. It invariably causes significant emotional arousal with associated numbness and denial. Many have acute stress reactions and some go on to have adjustment disorders and grief reactions;[43] A few patients have been diagnosed with post traumatic stress disorder as well.[44]

As this phase wears off, several psychiatric conditions can arise during the course of illness. Depression is the most common disorder amongst HIV positive patients with various studies reporting rates between 5-25%.[43] Anxiety disorders (phobias, generalized anxiety disorder, panic disorder) go hand in hand with depression and a study in a tertiary care unit in India has recorded that 40% of its patients suffered from depression while 90% of them fulfilled diagnostic criteria for an anxiety disorder.[45]

New onset of psychosis following HIV infections has been reported with rapid deterioration of mental and cognitive abilities. Risk of mania is also increased in HIV positive population than in general population. AIDS associated dementia is also a recognized entity which has a high prevalence in west but a low prevalence in India. Some attribute it to low level of detection and low life expectancy of patients in subcontinent while some argue there is a true reduction in prevalence.[43]

Side effects of treatment can have psychological manifestations as well. Many of these patients receive polypharmacy with room for many drug interactions. Psychological side effects of antiretroviral therapy range from anxiety disorders to psychotic episodes.[43] On the other hand, elevation of mood and better life satisfaction were reported in patients on HAART with improvement of quality of life.[46]

Very few studies are available on suicide and deliberate self harm in patients with HIV in the subcontinent.[45,47] Female sex, poor education, poor economic standing and degree of physical symptoms were positively associated with suicidal ideation.[47]

### Social

In the current social background of South Asia, a ‘label’ of HIV will be an enormous burden on the patient and family. It is seen as a disease of the sexually promiscuous. The patient and family face social isolation and ostracism. Despite many media campaigns and educational programs, the plight and prospects of the average HIV positive patient is still poor. On the whole, this marginalization as ‘us’ and ‘them’ will cause a significant problem in control of disease as people shy away from voluntary disclosure and testing. On preventive aspect, cultural taboos on discussion of sex and HIV, retard implementation of educational programs. Even languages in the region lack the vocabulary to address sex related issues. Therefore the use of mass media in preventive aspect is limited. Some authors maintain that specific communication models specifically targeted at high risk groups would be more effective in educating and initiating behavioral change.[48,49]

## HAART AND SOUTH ASIA

South Asia can be considered a resource limited setting with regard to management of HIV, cost of HAART is a major limiting factor in continuing drugs. However, the price of some first line antiretrovirals has reduced by 37 - 53% over the last decade. World Trade Organization (WTO) agreement on trade-related aspects of intellectual property rights has been modified by consensus allowing countries to produce generic drugs of HAART.[50] In India, a regimen of first line antiretroviral therapy (generic drugs) is estimated to incur a monthly cost of US$ 26.[40] Earlier when proprietary drugs were in the market, the annual cost of such a regimen was around US$ 20,000. Safety, efficacy and tolerability of generic HAART drugs have been established in studies in India.[40]

Even with reduced prices, governments find it difficult to cater to needs of all patients. For example, the gross annual per capita production in India is US$ 620 (2007) which makes the average Indian less able to afford HAART. Of all AIDS related expenditure, the single largest component is for drugs (40%).[40]

The system for providing drugs to patients differs from country to country. For example, in Sri Lanka, the government imports drugs and provides it free of charge to patients through state sector but in India such assistance is limited and a significant proportion of patients are left to buy drugs for themselves. Overall, the percentage of antiretroviral therapy coverage in the region is not satisfactory [Table 3].

**Table 3:** Estimates of patients on antiretroviral therapy in four countries of South Asian region[9,16,17,27]

Country	No. of patients on ART	No. of patients needing ART	Percentage of coverage (%)
India	158,000	Not known	Not known
Sri Lanka	<200	780	14-25
Nepal	1,400	20,000	7
Pakistan	600	20,000	3

Estimates not available for other countries of the region, ART - Anti retroviral therapy

The treatment problems which we need to tackle in future include; increasing coverage of antiretroviral therapy, ensuring a continuous drug supply, prevention of emergence of resistance by adhering to prescribed therapies (cost is the major factor affecting compliance), more public funding for HAART, lowering cost of second line HAART (5-8 times more expensive than first line therapy) and preventing inequalities to access of treatment at community and family level.

## SUMMARY

The South Asian region still has a low prevalence for HIV. However, the highest number of people with HIV outside Africa resides in India. The commonly accepted mode of transfer of infection in the region is heterosexual intercourse. Recognized high risk groups in the region include, female CSWs, IVDUs, MSM and migrant workers. Governments of all countries have perceived the threat and mobilized campaigns with patronage of both government and non government organizations to tackle the issue. The success of these programs varies from country to country and also dependent on political stability, political commitment, gender norms, religious and cultural restrictions and availability of funds. Though the cost of HAART has come down recently, the overall expenditure for patients with HIV is still a burden on the health care budget for all countries. In countries where data is available, the coverage of antiretroviral therapy is unsatisfactory.

The importance of maintaining the low prevalence status of the region cannot be over emphasized. Governments should consider funding more programs on preventive aspects as this would be a future investment. The social, cultural and humanitarian implications of a possible HIV epidemic in the subcontinent would be massive.
